# Industrial Hemp as a Potential Nonwood Source of Fibres for European Industrial-Scale Papermaking—A Review

**DOI:** 10.3390/ma16196548

**Published:** 2023-10-04

**Authors:** Dariusz Danielewicz

**Affiliations:** Papermaking Fibrous Pulps Technology Team, Centre of Papermaking and Printing, Lodz University of Technology, Poland, Wolczanska 223 Street, 90-924 Lodz, Poland; dariusz.danielewicz@p.lodz.pl

**Keywords:** industrial hemp (IH), kraft pulp mills (KPM), causal aspects, cultivation aspects, technological aspect, application aspects

## Abstract

The suitability of industrial hemp (IH) as a source of fibres for European industrial-scale papermaking, including, in particular, European kraft pulp mills (EKMPs) (i.e., plants producing the predominant amount of virgin pulps in Europe), was discussed, considering the causal, cultivation, technological, and application aspects of this issue. The work showed that there are generally premises for using straw from nonwood crops in European papermaking. As for the IH, it was found that IH stalks are the best IH fibrous raw material for EKMPs. There are a few cultivation factors favouring the use of IH stalks in them and a few, though important (e.g., small cultivation areas), factors not conducive to this use. Most technological factors favour the use of IH stalks in EKPMs, apart from the large differences in the length of the IH bast and woody-core fibres. The analysis of application factors indicates lower usefulness of IH stalks than wheat, rye or triticale straws, stalks of *Miscanthus × giganteus*, Virginia mallow, and kenaf. This is due to the much greater availability of these cereal straws than IH and less variation in the fibre length of cereal straws, *Miscanthus × giganteus*, Virginia mallow, and kenaf than in IH stalks. The main conclusion from the conducted query is the statement that the presence of IH varieties with fibre lengths more similar to wood would reduce the number of technological and application factors unfavourable to their use in EKPMs and increase the competitiveness of hemp straw vs. wood as a raw material for European large-scale papermaking.

## 1. Introduction

Manufacturing products from synthetic polymers and the combustion of fossil fuels has caused a large amount of pollution in the natural environment (non-biodegradable solid waste and harmful gases from illegal garbage incineration) and greenhouse gas emissions (e.g., CO_2_) worldwide. For this reason, there is currently an increased interest in using raw materials, semi-finished products, chemicals, and biofuels of plant origin in different industries [1]. The substitution of synthetic polymers in products made in these industries may be partial or complete. For this aim, non-processed natural fibres, plant meals, starch, and primary fibrous papermaking pulps can be used [2,3,4]. As for primary papermaking pulps, they are currently used most often for producing some kinds of papers and paperboards. These intermediates are also used for the production of substitutes for products made of plastic, such as form-moulded pulp products (e.g., three-dimensional packaging for eggs, etc.) [5], viscose and cellulose acetate fibres, cellulose films, and various types of cellulose derivatives. They can also serve as a source of simple sugars, which can then be converted into bio-alcohols and bio-chemicals [6,7]. This is possible because the main components of cellulose pulp include polysaccharides (as cellulose and hemicelluloses) and a fibrous form that makes their saccharification much easier than even finely chopped wood.

**Figure 1:**
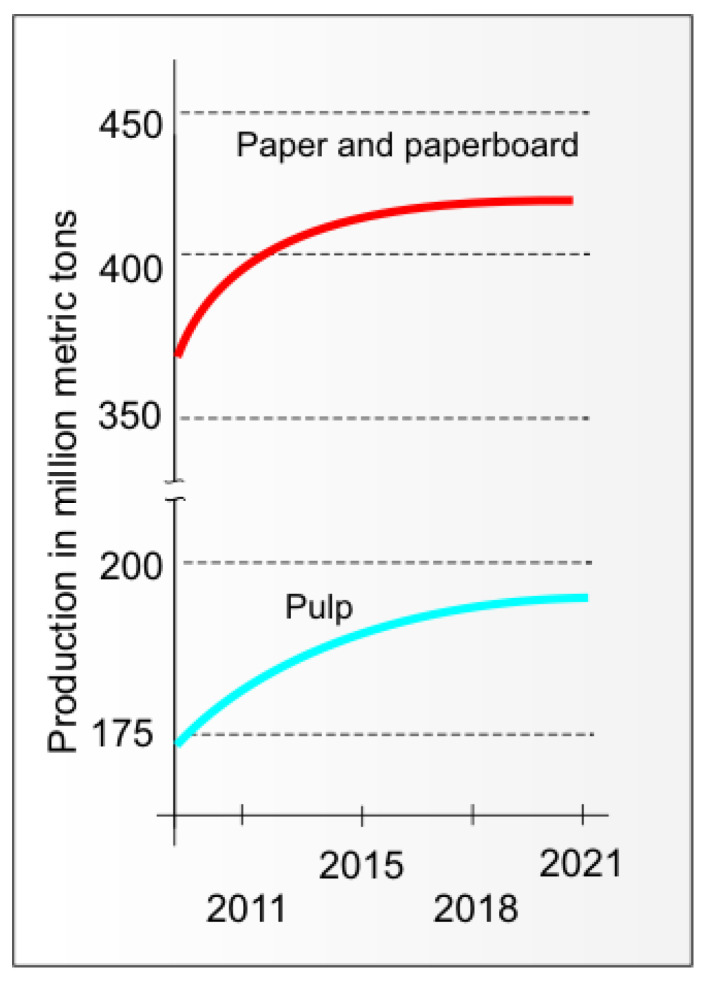
Production of pulp (blue line), paper, and paperboard (red line) in the years 2009–2021 (artwork of the author based on [8]).

For many years, the continuously growing production of paper and paperboard and the trend of increasing the number of market products manufactured using papermaking pulps other than paper and paperboard meant that their production did not decrease between 2009 and 2021, according to [8] (Figure 1), despite the decrease in the production of graphic papers as a result of the increasing popularity of electronic information transfer techniques.

Continuing the high production of pulp and paper products along with the growing demand for wood from other industries, together with the disapproval from public opinion and the media regarding the harvesting of wood from natural forests for economic purposes, and demanding the exclusion of more and more forest areas from normal forest management (e.g., by the European Commission because of the need to protect biodiversity and the link of deforestation with greenhouse gas emissions [9]) collectively mean that the pulp, paper, and wood particle board industries located in Europe will have to consider the use of fibrous production intermediates manufactured from nonwood fibrous raw materials (NFRMs) [10,11]. This trend is also supported by the following facts:

− So far, fibrous raw materials (FRMs) have been used in these industries to a relatively small extent and constitute a fairly large FRM base.
− They are much better suited for use in the pulp and paper industry than, for example, in the furniture or construction industries.
− The waiting period for straw from nonwood plants is much shorter than that for wood.
− In some cases, they show a higher CO_2_ absorption capacity per ha of cultivation than woodlands [12].
− Substituting some of the wood with nonwood fibres would make it possible to switch some of the papermaking production to papermaking products with reduced content of wood fibres from natural forests, which could be given a special trademark.

Taking this into consideration, a review of the literature on various aspects of the possible use of one nonwood raw material, industrial hemp (IH), in European industry-scale papermaking, for which pulp is mainly produced in kraft pulp mills (EKMPs), was conducted, considering the following aspects: causal (Section 2), cultivation (Section 3), technological (Section 4), and application (Section 5) [13]. 

The data collected in the work and the conclusions drawn regarding this issue may be useful for the following:

− Managers and engineers working in large European pulp and paper factories interested in the idea of producing paper products with a reduced content of fibres from natural forests;
− Farmers interested in the cultivation of hemp intended for seeds and inflorescences, who see hemp straw for papermaking for the possibility of its more rational and less burdensome use than burning and its separation into bast fibres and shives, respectively;
− Research centres breeding new varieties of industrial hemp, characterised by better functional properties.

## 2. Causal Aspects

### 2.1. The State of the FRM Base in the European Pulp and Paper Industry and the Possibilities of Its Expansion

Forests are a traditional source of wood for papermaking around the world. The surface area of the forest land, according to FAO data on a global scale, is decreasing every year [14]. This is because of poor forest management in many countries, the transformation of forests into agricultural lands, forest fires, and damage caused by insects [15,16]. For this reason, there is an increasing interest in acquiring wood for papermaking from forest plantations for industrial processing. However, the possibility of obtaining wood from this source is greatest in countries with a warm climate (in Europe, mainly Spain and Portugal) [17,18,19]. In countries located in the northern temperate climate zone, there are shortages of cheap wood for papermaking [9,20,21]. In order to increase the competitiveness of the pulp and paper industries in these countries, forest plantations are also planned, in which poplars, spruces, larches, and birches are cultivated [22,23]. An example is the plantation of fast-growing poplar hybrids set up in 2012 in Poland by International Paper in cooperation with GreenWood Resources. The area of these plantations and, therefore, the amount of pulpwood that could possibly be obtained is small, however, and the quality of the wood is uncertain, so it may even be necessary to use it for heat production at pulp mills, as speculated by Duda [24].

Since primary papermaking pulps are expensive, many companies use waste paper instead to make paper and cardboard. This papermaking FRM, because of the development of technology and techniques for upgrading waste paper, can also be processed into relatively good-quality pulps [25,26]. In 2010, this type of FRM accounted for approximately 45% of the raw material base of the paper industry in the world. However, there is also a shortage of waste paper because of the increasing number of companies using it for the production of papers and paperboards, as well as due to the reduction in the amount of graphic paper production [27].

NFRMs are used in a relatively small amount for papermaking globally (up to approx. 8–10% of papermaking pulps were produced from this kind of FRM in 2010) [28]. The main reason for this was the easier production of pulp from wood than from the straw of nonwood plants. This is because wood is easier to transport, more durable in storage, and enables one to obtain more balanced and better paper quality. The most important NFRMs used in the production of paper and cardboard in the world are mainly straws of wheat, rye, triticale, rice, bagasse, and bamboo wood [29,30,31]. For example, in the years 1996–1998, these materials accounted for about 68% of all the NFRMs consumed globally for papermaking (cereal straw 46%, bagasse 14%, and bamboo 6%). The remaining part of the nonwood pulp and paper is made from reeds, cotton linters, esparto, flax, hemp, kenaf, jute, abaca, and sisal fibres [29,32].

Most of the listed species of nonwood plants used in the world for papermaking, however, do not grow in European countries located in the northern temperate climate zone. Therefore, in these countries, for papermaking, mainly those plants that grow well in this climate zone can be considered such as cereal straws (e.g., wheat, triticale, and rye). However, an unfavourable feature is their high content of mineral substances and, in most cases, the high density of the paper produced from cereal straw pulp. For this reason, nonwood plants that grow well in the temperate climate zone, such as industrial hemp [33,34,35,36], should be considered. 

### 2.2. The Long Tradition of IH Cultivation in Europe and the Multiple Uses of this Plant in the European Economy

Hemp (*Cannabis sativa* L.) was one of the first plants commonly grown by humans to obtain fibres for fabric production. It is characterised by rapid growth in the northern temperate climatic zone; moderate weather and soil requirements; the capability of growing without the use of large amounts of pesticides and herbicides; and resistance to rodents. The earliest records of the use of this plant originate from Japan and China (years 4500–7000 BCE), from where, probably because of population migration, it first reached the Caspian Sea region and then ancient Rome and all of Europe, becoming widespread after 500 BCE. After the discovery of America, it also found its way to that continent, where it was widely cultivated until the 1930s. After this period, the size of the crop area decreased relatively quickly. This was because of the widespread cultivation of cotton; the import of cheaper fibres (abaca, jute) from Asia; the intensive development of technology for producing artificial fibres; and the ban introduced on IH cultivation because of the possibility of producing narcotic substances from it [12,37,38].

Currently, because of the return to the trend of manufacturing industrial products using natural raw materials, there is increased interest in the cultivation of fibre plants, including IH [39]. IH can be used for the production of textiles, haberdashery, and footwear [12,40,41,42,43]; building materials [42,44,45,46]; composites (e.g., car parts) [42,47,48]; energy, heat, and fuel [49]; cosmetics and medicines [42,50,51,52,53,54]; food (oil, flakes, margarine) [55]; paints and varnishes [46]; ropes and twines [12]; as well as papermaking pulps and products [12,56,57]. There is also the possibility of using IH to remove heavy metals from soils contaminated by industry [43,58]. An incentive to increase IH cultivation and processing is also the establishment of subsidies for hemp cultivation in some EU countries [33,59,60,61].

As it turns out, it is most profitable for farmers to cultivate IH for inflorescences and seeds to obtain essential oil and food products, respectively [4]. According to Czapluk and Czerniak [55], in Poland, 82% of farmers’ income from IH cultivation came from dried inflorescences, 13% from seeds, 3% from bast fibres, and 2% from IH straw. The seeds can be sold, used for sowing IH in the next year, or processed into food raw materials, like oil, flour, and protein powder, while inflorescences serve as a source of active substances that are then used to make medicines, dietary supplements, and cosmetics [42,43,51,52,62,63]. According to Burczyk et al. [52], the amount of inflorescences that can be collected per ha of IH cultivation depends on the sowing density and harvesting period and is usually 1–4 t, while the seed yield is about 1–1.5 t. Here, however, the problem arises from the most rational and simultaneously uncomplicated management of hemp straw, other than its burning or quite complex division into bast fibres and hurds called decortication.

The above fact creates an opportunity for the papermaking industry to obtain IH straw for its needs. However, it is possible, provided that hemp as papermaking FRM will be an attractive FRM in terms of availability, price, and properties in comparison to other NFRMs.

## 3. Cultivation Aspect

### 3.1. Definition of IH

Knowledge on the basic issues regarding the legality of IH cultivation is of great importance for assessing the possibility of its use in the pulp and paper industry. The facts for IH are as follows. IH is a one-year plant belonging to the genus *Cannabis sativa* L., in which the sum of the content of Δ-9-tetrahydrocannabinol acid (THC) in the dried upper fragments of stalks containing female inflorescences does not exceed 0.2–0.3% of their dry matter harvested in the green state during the flowering period (cultivation of IH with THC content in inflorescences higher than the range given is forbidden in many countries) [64]. In the years 2004–2011, Zachwieja [65] examined, annually, the THC content in Polish varieties of IH, which never exceeded 0.2% and most often was below 0.1%. However, the problem is that IH in the initial stage of development does not differ substantially from narcotic hemp. Such differences (shorter stalks and more branched inflorescences) appear only in the later phase of hemp stalk growth. For this reason, the exact identification of the type of industrial hemp can be carried out on the basis of the results of THC determination in the plant inflorescences [66,67]. Apart from that, the decision of the United Nations’ Commission for Narcotic Drugs to remove hemp from the category of dangerous drugs in 2020 [43] can be considered an important event from the point of view of reducing the reluctance of the pulp and paper industry to use IH straw as a raw material for the production of papermaking pulps and then papers and cardboards.

### 3.2. The Size of the IH Crop Area

For IH to be considered an FRM that could play a role in e.g., EKPMs, it should account for at least 1% of the FRM used to produce kraft pulp in a standard kraft pulp mill. As we know, such pulp mills are most often designed to produce about 1000 t of oven-dried (o.d.) pulp per day, which, with a 50% pulp yield from wood, requires a supply of 2000 t of o.d. wood per day. In this case, the annual demand for IH straw would be 320 × 2000 t of o.d. IH straw, which gives a demand of 640,000 t of o.d. IH straw annually, if hemp were to replace wood completely. This would require sowing 640,000/8 = 80,000 ha (assuming the possibility of obtaining only 8 t of o.d. IH stalks without leaves from one hectare of cultivation). Therefore, the area for IH cultivation in a given country is important. In the period before the Second World War, as well as immediately after it, the size of the cultivation area for IH was considerable. For example, in Poland, in 1964, about 15,000 hectares of arable land were allocated to IH cultivation. However, the size of the area of these crops in the twentieth century was gradually reduced because of the widespread use of synthetic fibres in the textile industry, as well as the increase in the profitability of growing cereals, and so in the years 2006–2012, it amounted to only 400–1000 ha [68], in 2018–2020 increased to about 3000 ha [69], and decreased again in 2022 to 1700 ha because of the increase in the prices of agricultural products, such as rapeseed, wheat, and corn [70]. 

As for the world, at least 70 countries cultivate IH for commercial or research purposes. The largest producers of IH are currently Canada, China, the USA, France, and Chile [43]. Lately, the IH acreage in North America and European Union countries amounted to about 30,000–46,000 ha and about 36,000–55,000 ha, respectively. However, on a global scale, the area of IH cultivation was at the level of approximately 200,000–220,000 ha [71,72]. In Europe, IH cultivation was centred in France (several thousand hectares), Germany, Lithuania, Estonia, the Netherlands, Romania, Italy, and Austria (in all of these countries, it amounted to several thousand hectares) [71].

Because of the legalization of IH cultivation with low THC content and the growing demand for products made from materials of natural origin, many farmers have recently started growing IH or expanded their cultivation. However, there are signs that the large supply of IH and the increasing profitability of growing cereals will result in a decline in the price of IH and a decrease in the acreage of this crop in some regions of the world. Other factors may also contribute to this phenomenon. These are more expensive and less soft textiles made from IH bast fibres compared to those made from cotton and synthetic fibres, the low content of CBD (cannabidiol) in IH, and the higher prices of IH products (e.g., oil). This suggests that, for now, one should consider IH as a special plant that cannot compete with cereals and that its market still needs several years to mature and stabilize [73].

### 3.3. IH Cultivars

In many countries in the world where the cultivation of IH has a long tradition, different varieties of this plant have been grown. These are, for example, the Hungarian varieties Uniko, Kompolti, and Fabriko; French: Fibrimon, Férimon, Fédor, Félin, Futura; Romanian: Secuieni, Irene, Lovrin, Fibramulta; Finnish: Finola and Anka; Russian: Kuban, Zenica, Dneprovskaya Odnodomnaya, Zolotonoshskaya, Yuso; Italian: Carmagnola, Fibranova, Eletta Campana; Yugoslavian: Novosadska konoplja, Spanish: Delta-405 and Delta-Llosa; Czech: Rastslaviska; and German: Fasamo [35,74].

In Poland, according to the national register of varieties of agricultural plants kept in the Research Center for Cultivar Testing (COBORU) in Słupia Wielka, in 2014, varieties registered included: Beniko, Białobrzeska, Rajan, Tygra, Wojko, and Wielkopolskie [75]. 

However, these varieties of IH should be considered as varieties whose main purpose of cultivation is to obtain the largest possible amount of bast fibres for the needs of the textile industry, the largest possible amount of biomass of IH stalks, and the best quality of bast fibres from the point of view of their suitability for the production of clothing.

It is worth noting that, recently, a need has also been identified for IH varieties capable of yielding more IH seed oil and inflorescences than the previously known cultivars. For example, these include the oily variety Henola, which enables one to obtain more seeds than the fibrous IH cultivar Białobrzeskie and a variety of unknown names obtained in Italy by the start-up Aeroponica Perrotta, allowing for the production of significantly more CBD than regular IH varieties provide from inflorescences and roots. Unfortunately, from the point of view of papermaking, a common, unfavourable feature of these varieties is their shorter stem and the related lower yield of biomass per hectare of cultivation [63,75,76].

As for varieties of IH specially bred for the needs of the pulp and paper industry, the number of reports is limited. Burczyk [77] was the only one to report that the Institute of Natural Fibres and Medicinal Plants in Poznan recently developed a variety of IH that has a 12.5% higher cellulose content than the average straw of the Białobrzeskie variety and a high yield of biomass per hectare. 

### 3.4. Yields of IH Stalks per ha of Cultivation 

The anatomical parts of IH that can be used in papermaking are the stalks, woody core, and bast fibres. The amount of dry IH stalks that can be obtained per hectare of cultivation increases with the amount of their dry biomass (i.e., stalks plus leaves). The total amount of dry IH biomass from one ha of cultivation has been determined by many authors [33,34,36,61,78,79,80,81,82,83,84,85,86,87,88]. The yield of dry IH stalks, however, is reported by only a few of them, namely Struik et al., Pahkala et al., Cosentino et al., and Angelini et al. [33,34,36,61]. The research by these authors shows that the amount of dry IH stalks that can be obtained per hectare of cultivation varies from 5 to as much as approx. 22 t per year, depending on the variety of IH; the region in which the crop was located; the fertilization intensity; the soil type; and the amount of rainfall. For comparison, the annual increase in the volume of spruce trees, European larch, Douglas fir, birch, and various species of genetically modified poplars per 1 hectare of plantations in Poland amounted to approx. 4–5.2 t, 3.2–5.5 t, 5.4 t, 2.9 t, and 5.7–12.3 t of dry wood [22], respectively. In turn, the amount of dry wheat straw together with leaves and spikes that can be obtained per ha of cultivation in Polish conditions is 2–7 t [84,89].

In this respect, IH can be competitive in relation to spruce, larch, Douglas fir, birch, cereal straw, and genetically modified poplars if the dry biomass annual yield per hectare is higher than 7 t and 6–12 t, respectively. 

## 4. Technological Aspect

### 4.1. Material and Bulk Densities

One of the significant disadvantages of NFRMs is that they have lower material and bulk density than wood, which reduces the amount of FRM that can be transported to kraft pulp mills at one time on a car or rail platform and the amount of FRM that can be loaded into a digester. A comparison of the densities of these types of cut IH stalks with the densities of pine, birch, and wheat straw has been presented in the literature [90]. It turned out that the material density of IH stalks was significantly higher than wheat straw and slightly lower than pine. On the other hand, the bulk densities of IH stalks with and without load (500 g) are similar to those of pine chips, whereas the values of these indices of wheat straw are significantly lower than those of pine chips.

### 4.2. The Content of Chemical Components in Anatomical Parts of IH Stalks 

The content of chemical components in IH should be considered in relation to its different anatomical parts, namely stalks, bast, technical fibres, and woody core. Data enabling the comparison of the content of individual chemical components in these parts of IH stalks are presented in Table 1.

Comparisons of the chemical composition of IH stalks of the Polish variety Białobrzeskie without inflorescences and leave, the woody core of this variety separated from stalks not subjected to the retting process, and technical bast fibres, as well as birch and pine determined with the same analytical methods, are shown in Table 2 [101].

The data in Table 1 and Table 2 show that the individual anatomical parts of IH have different contents of chemical components. IH bast has the highest content of cellulose, followed by IH stalks and woody core, and the highest lignin content is in woody core, while the lowest is in IH bast. In terms of the content of cellulose and lignin, IH stalks are a better material for the pulp and paper industry than pine and birch wood. Table 2 also shows that in the selected (without inflorescences and leaves) IH FRMs, the content of mineral substances is 1.2–1.5% of their dry matter.

### 4.3. Fibres of IH Stalks

**Figure 2:**
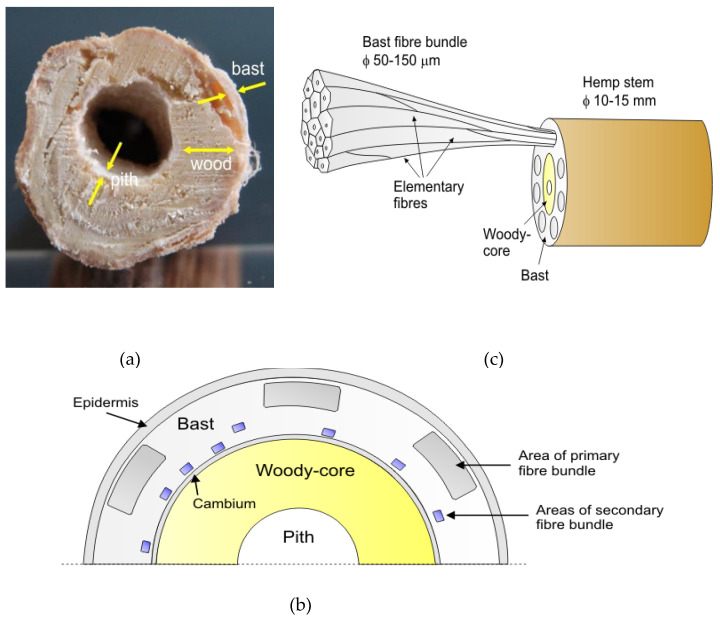
Cross-section of the IH stalk [101] (**a**), distribution of bundles of primary and secondary bast fibres in the cross-section of hemp stalk (**b**) [101,102,103,104], as well as the structure of IH technical bast fibres (**c**) [102] (**c**) (**b**,**c**—author’s drawings based on [101,102,103,104]).

In addition to the biomass yield per hectare of cultivation and the content of individual chemical components, the kind and dimensions of the fibres found in IH are of considerable importance to the papermaking industry. As mentioned, the basic elements of the anatomical structure of the IH stalks are bark, woody core, and pith, the latter lining or filling the hollow core canal of the stalk (Figure 2a).

The bark, whose share in the IH stalks is from 30 to 35% by weight [78] is composed of peel (epidermis) and bast (phloem). Bundles of primary bast fibres are distributed in the outer part of the bast and are surrounded by parenchyma cells, while the bundles of secondary fibres are located closer to the woody–core of the stalk and are surrounded by the parenchymatous–conductive tissue (parenchyma cells and sieve tubes) (Figure 2b) [104,105]. Fišerova et al. [96] report that the primary bast fibres are much longer and have a lower lignin content compared to the secondary fibres.

Bast fibres, unlike wood, are multi-cell formations built of a large number of elementary fibres (Figure 2c) [103,104]. The binding substance of these fibres is a natural resin (vegetable gum), the key components of which are pectin as well as lignin, hemicelluloses, and polyuronides [40,104,105,106,107,108]. Data on the morphological properties of IH technical fibres were presented by Cierpucha et al. [40]. According to these authors, the length of these fibres ranges from 80 to 300 cm (usually 120 cm), while the length of IH elementary fibres ranges from 5 to 55 mm (usually 15–25 mm). As mentioned above, in addition to the appropriate primary fibres, the bark of the IH stalk also contains a certain number of fibres that are of little use for textile purposes, called secondary fibres. Unlike the primary fibres, they are shorter, wider, more intertwined, and more fragile [40]. According to Lisson et al. [108], the length of these fibres is about 2 mm, while their width is about 17 μm, i.e., about 50% less than primary fibres. The secondary fibres are formed from the moment of the creation of flower buds to their maturation stage (so later than the primary fibres) [66]. Most of these fibres occur in the lower part of the IH stalk [66,103]. Their share in the phloem is 10–45% [78,96,104].

Separating the elementary fibres from the technical fibres is difficult because of the substances that bind them together [109,110]. However, the degradation of these substances in the processes used in pulp mills, such as kraft pulping, should not pose major difficulties.

The IH stalk contains two more layers, i.e., the IH woody core and pith (Figure 2b). From the point of view of its use in papermaking, the IH woody-core layer has a certain importance. In the process of extracting bast fibres, the woody inner layer, after breakage, is referred to as shives or hurds. IH shives, because of their different colour and content of mould, IH fibres, leaves, and dust, are available on the market under the names “white shives”, “grey shives”, “fibred shives”, and “defibred shives” [108]. One should, therefore, take account of the possibility of their various usefulness for papermaking. The IH woody-core layer is made of IH wood fibres (libiforms), vessels, and parenchyma cells [104,105]. The pith of the IH stalks is made of parenchyma cells. In the wide part of the stalks, the cells of the core dry up and cover the core channel wall with a thin layer; in the stalks of young plants and the upper parts of mature plants, they can fill the entire interior of the stalk [105].

**Figure 3:**
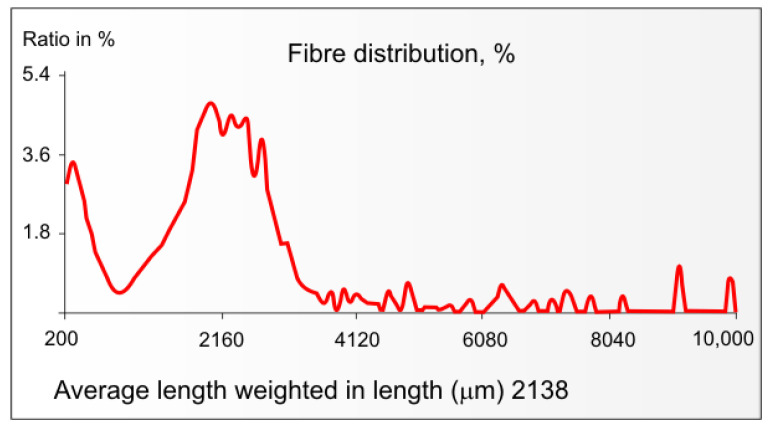
Fibre distribution of pulp from IH bast (author’s drawing based on author’s results from MorFi apparatus, TechPap).

Figure 3 shows the distribution of fibre lengths in soda-oxygen pulp from IH bast mechanically separated from hemp woody core [101].

**Figure 4:**
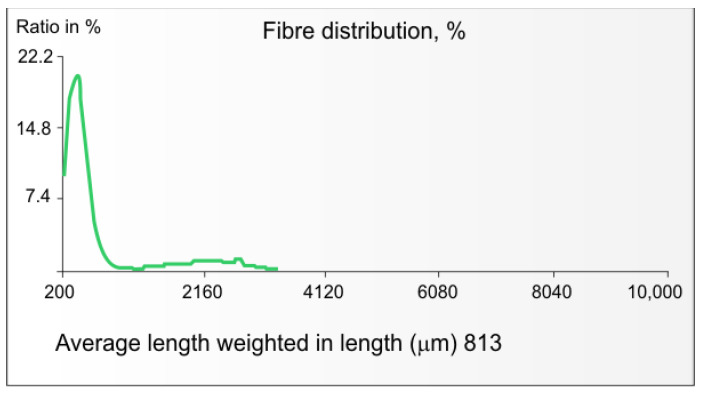
Fibre distribution of kraft pulp from IH stalks cut into 0.3–0.6 mm sections (author’s drawing based on author’s results from MorFi apparatus, TechPap).

As seen in Figure 3, in the range of fibre lengths of 0.2–10.0 mm, the IH bast pulp contains a fraction of fibres with a maximum length of about 0.2–0.3 mm, a fraction of fibres of medium length (0.5–4.1 mm) of probably secondary bast fibres, and a fraction of long bast fibres with a length of 4–10 mm and more. Figure 3 contains no data regarding the content of long bast fibres in the IH bast pulp with a length between 10 and 55 mm. This is because the measuring range of the MorFi apparatus is 0.2–10 mm [111]. Because of their length, these bast fibres can pose challenges in the papermaking process, causing issues with spinning, stopping on the sieves of sorting devices, and paper formation. The solution to the problem of the presence of 10–55 mm hemp bast fibres in hemp stalk pulp is to cut the hemp stalks into appropriate sections before pulping. The effect of cutting these stalks into such sections on the distribution of the fibre length in the kraft pulp is presented in Figure 4.

Figure 4 shows that the pulp from IH stalks cut into 3–6 mm sections does not show detectable amounts of bast fibres longer than 4 mm and that the average fibre length of the pulp is approx. 0.8 mm. There are two types of fibres shown in this figure, i.e., the IH woody-core fibres (on the left) with an average length of 0.5 mm and the bast fibres (on the right) with an average length of 2.2 mm. As for the average length of hemp woody core, similar values (0.5 mm) have been reported by Correia et al., de Jong et al., and Lisson et al. [91,94,108].

### 4.4. Pulping Methods and Their Effects (Yield of Pulp and Pulp Properties)

A review of the papermaking literature shows that IH FRMs (stalks, phloem, and woody core) can be processed into fibrous papermaking pulps using many pulping methods, such as soda [78,91,112,113,114,115,116]; soda anthraquinone (Soda-AQ) [96,117]; soda-oxygen [92,115]; soda- or potassium-peroxide [115,118]; kraft [91,99,100,115,117]; kraft with the addition of anthraquinone (Kraft-AQ) [115]; kraft-NaBH_4_ [98]; alkaline-sulfite [115]; neutral-sulfite [119]; organosolv [93,120,121]; thermomechanical and cold-soda (CS) [108]; alkaline-peroxide (APXP) [94]; chemithermomechanical [95,122,123]; and sulfite [92,115,118]. Of these methods, however, attention should be paid to kraft pulping. According to the CEPI report [13], in European countries associated with this organization, the share of kraft pulp production in total pulp production and the production of chemical pulps were 73.0% and 94.2%, respectively, and, therefore, it was dominant.

As for the total yield of pulps from IH stalks and hemp woody core containing low contents of residual lignin (bleachable grade, kappa number ~20) and sack grade pulps (kappa number ~50), the review of papermaking works indicates that it is between 56–70% and 40–52%, respectively [91,93,96,100,112,114,115], while the yield of pulps made of IH bast fibres is reported to be higher, i.e., between 60 and 85% [93,100,115,118]. 

**Figure 5:**
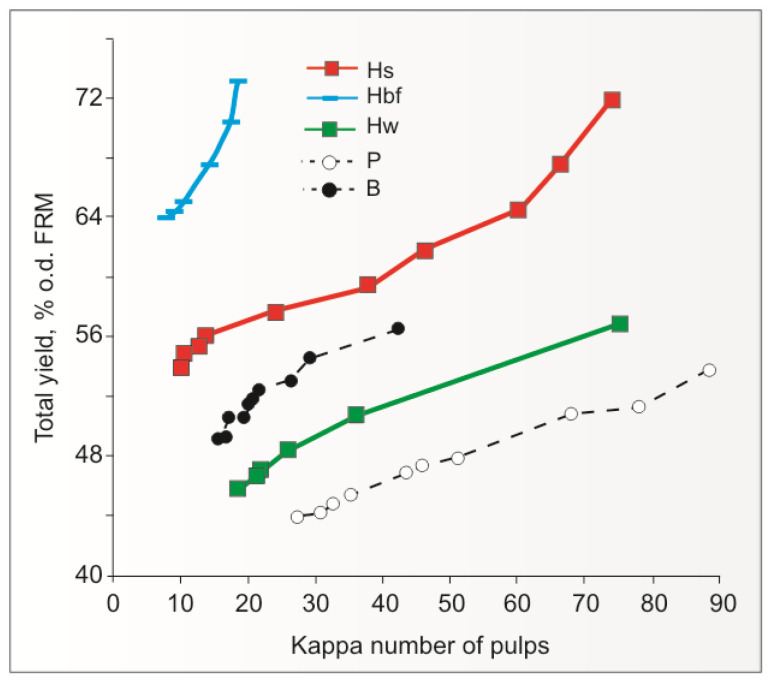
Digester (total) yield of unbleached kraft pulps from: IH stalks (Hs) and IH woody-core (Hw) kraft pulps, birch (B) and pine (P) kraft pulps; and alkali-oxygen ECF bleached IH bast fibres pulp (Hbf) [101] (author’s drawing based on author’s results).

The issue of the yield of unbleached kraft pulps from IH FRMs (stalks, woody core, and bast fibres) is well illustrated in Figure 5. 

These data indicate that by subjecting IH stalks (Hs), woody core (Hw), and bast fibres (Hbf) from the Białobrzeskie variety to kraft pulping, it is possible to obtain bleachable-grade and sack-grade kraft pulps from these FRMs, with a yield of 46–54%, 56–64%, and 64–74%, respectively, in their KN range of 20–60 units. 

The yields of ECF (Elemental Chlorine-Free) bleached kraft pulps from IH stalks, woody core, and bast fibres were also determined. The first one turned out to be higher than the yield of bleached birch kraft pulp by 5%, the second one was higher than the yield of bleached pine kraft pulp by 3%, and the third one (pulp obtained from IH bast fibres through its oxygen delignification and ECF bleaching) was higher than the mentioned birch (B) and pine (P) kraft pulps by 37 and 43%, respectively [101,124].

**Figure 6:**
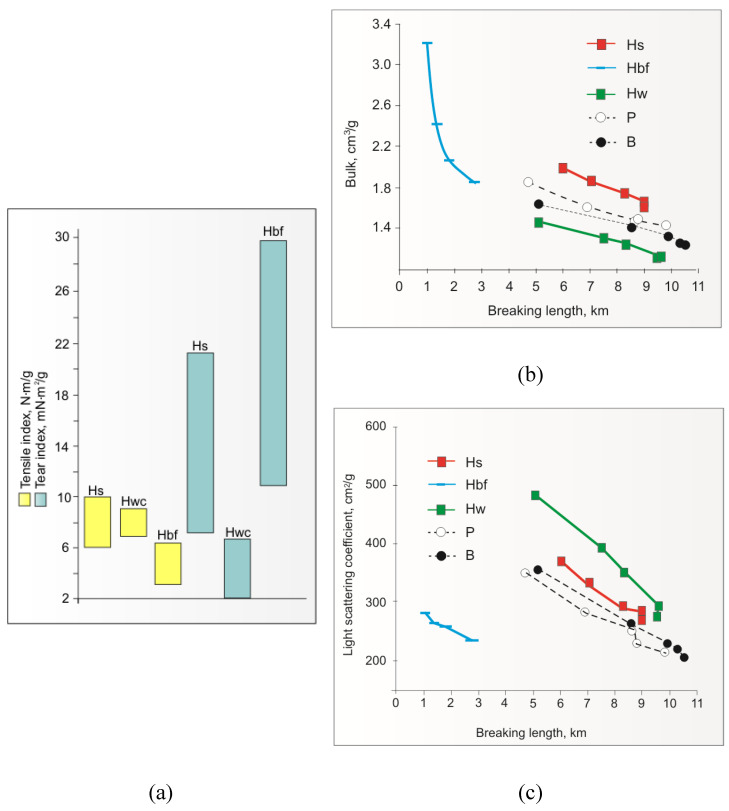
Comparison of the tensile (**a**), tear index (**a**), bulk (**b**) based on results presented in [94,96,98,114,115,125], and bulk and light scattering coefficient (**c**) of handsheets of paper prepared from unbleached and bleached Hs, Hw and Hbf pulps (author’s drawing based on author’s results [101]).

As for the strength properties of IH pulps, studies showed the possibility of achieving a medium or very good (60–100 N⋅m/g) level of tensile strength of handsheets made from IH stalks (Hs) pulp [93,96,97,114,115,117], very good (70–90 N⋅m/g) tensile strength level of IH woody-core (Hw) pulp [113,116], and a low or medium (30–65 N⋅m/g) tensile strength level of IH bast fibres (Hbf) or IH bast pulps [93,115,125] (Figure 6a, yellow columns). 

Also interesting is the resistance to the tearing of handsheets for pulps from individual anatomical parts of IH stalks. The values of this characteristic of IH bast fibre pulp or IH bast fibre pulp are high (11–30 mN⋅m^2^/g) [93,125], while for handsheets made from IH woody-core pulp, they are very low (2–6 mN⋅m^2^/g) [93,113,125]. This characteristic of IH pulps from IH stalks has intermediate values, but for moderate beating, they may also be high (7–21 mN⋅m^2^/g) [96,125] (Figure 6a, blue columns).

Other noteworthy features of IH kraft pulps that are important for the quality of paper are the high bulk from IH bast fibres or IH bast pulps; the good bulk of IH stalk pulp (comparable to the bulk of pine kraft pulp) (Figure 6b); the good light scattering coefficient (opacity) of IH woody-core pulp and IH stalk pulp (Figure 6c); the rapid increase in tensile strength of IH woody-core pulp; the semolina-like consistency of the suspension of this pulp; the high content of α-cellulose in the ECF bleached pulp from IH bast fibres; and the possibility of the negative influence of the presence of IH bast fibres in the fibre composition of paper on its formation [125].

As it turned out, the properties of IH pulps can be explained well using the values of the length of the bast and woody-core fibres, the number of these fibres in pulps, and the flexibility/conformability index (F/C I) calculated by dividing the fibre width by its cell wall thickness (Table 3) [101].

## 5. Application Aspect

### 5.1. Compatibility of Devices Used in EKPM with IH FRMs and IH Pulps

A relatively small number of examples on the subject of making pulp and paper from IH on an industrial scale in Europe is available in the literature. They discuss the use of IH bast fibres for the production of special or fine-quality papers (e.g., cigarette paper) [56,126] and IH stalks for the production of bleached pulp, which is probably used for the production of similar types of products in the UK, USA, Germany, France, and Spain [57,127]. No data are available on the specific features of the equipment used in pulp and paper mills for processing IH FRMs into pulp and producing paper using IH pulps. Although the functioning of papermaking plants producing niche hemp papermaking products proves that it is possible in industrial conditions to process much longer or much shorter IH fibres in pulp and paper devices, it should be expected that in large-scale EKPM prepared for the processing of wood fibres, the following problems may occur when long IH fibres appear in the production system:

− Clogging of the extraction sieves of the digesters by IH bast fibres;
− Spinning of these fibres on rotating elements of pumps, defibrators, mixers, etc.;
− Clogging of the sieve openings of screen sorters and washing devices;
− Hindering the circulation of the pulping liquor in the chip column of the digester.

Certainly, one of the possibilities for solving these problems is the production of pulp and paper from mixtures of wood and properly cut IH stalks, with a relatively small share of these stalks in the mixture of FRM. This causes too long and too short IH fibres to be dispersed in the suspension of wood fibres [128,129]. 

However, a more rational solution seems to be growing a variety of IH with a significantly lower length of fibres in the bast (e.g., as in kenaf) and somewhat higher (e.g., by 0.15–0.3 mm) in the IH woody core (e.g., such as in eucalyptus wood).

### 5.2. Comparison of the Ease of Processing IH FRMs into Kraft Pulps 

The lignin content in IH stalks suggests that it would be easier to process them into kraft pulp than many kinds of pulpwood because it is lower in the case of IH stalks. Research results confirm this statement because IH stalks can be pulped into 25 kappa kraft pulp with approximately 15% active alkalis dosed on o.d. FRM, while birch wood requires approximately 18% of these alkalis on o.d. wood [100]. 

Considering the ease of processing the IH woody core into kraft pulp based on its lignin content (approx. 26%), it can be considered an intermediate between hardwood and softwood, with a shift towards hardwood due to the possible better susceptibility of IH lignin for delignification, due to its nonwoody nature (as a rule, nonwood FRMs can be pulped easier than wood) [100]. 

Due to their low lignin content, IH bast fibres are the IH FRM with the greatest ease of processing into papermaking pulps because only oxygen delignification and bleaching can be used for their processing into such pulps [100]. 

When considering the ease of processing IH stalks and bast fibres into kraft pulps in EKPMs, one should also take into account the need to cut these FRMs more finely to shorten the length of the bast fibres, as opposed to the usual cutting into 2–3 cm sections of cereal straw, *Miscanthus* × *giganteus*, Virginia mallow, or kenaf. This may make processing IH more difficult than processing the listed alternatives to IH.

### 5.3. The Effect of Replacing Part of the Wood with Hemp Raw Materials on the Yield of Kraft Pulp and Paper Properties

In terms of yield, IH kraft pulps from IH stalks can be included in the group of FRMs, which give unbleached kraft pulp with a total yield similar to that of European aspen (*Populus tremula*) unbleached kraft pulp and, therefore, one of the highest among kraft pulps from wood (approximately 57% at KN of pulp 20) [90]. Such a pulp yield from IH stalks is a significantly advantageous feature in comparison with pulps from classic types of wood from which kraft paper pulps are made, i.e., birch and pine (yields at the KN 25 are 53 and 44%, respectively) (Figure 4). This is due to the presence of IH bast fibres with a high cellulose content and a lower lignin content in the IH stalks. Therefore, replacing part of the wood with IH stalks should positively affect the pulp yield from the mixture of FRMs.

Data analysis of the subject literature indicates that IH stalk pulp can have the best papermaking properties among IH pulps [101] because its properties are at the level of pulps from some traditional pulpwood used in EKPMs [130,131]. For example, the bleached IH stalk kraft pulp obtained from IH stalks cut into 3–6 mm fragments is characterised by a tensile index similar to bleached pine kraft pulp, a somewhat higher tear resistance and bulk than bleached kraft pulp from wood, and a light scattering ability and opacity that are comparable to bleached birch kraft pulp after beating. These properties of IH stalk kraft pulp result from the dimensional characteristics of its fibres and its chemical composition. For example, the high ability of the fibres of this pulp to bond with each other (high tensile strength index) can be explained by the presence of thin-walled fibres in a woody core. In contrast, its high resistance to tearing can be explained by the presence of long and stiff IH bast fibres (Table 4). Comparing the average fibre length of IH stalk pulp from IH stalks cut into 3–6 mm fragments, one can state that it is higher than or comparable to the average fibre length of kraft pulps from certain acacia and eucalyptus varieties [132,133,134] and comparable to or somewhat lower than the average fibre length of birch kraft pulp [133,134].

It is worth noting that the resistance to tearing of the pulp from IH stalks can be programmed to a certain extent at the initial cutting stage of these stalks by using their shorter or longer sections for pulping. This may make it possible to obtain pulp whose tear resistance exceeds the value of this feature of pine pulp, which, in turn, creates the possibility of using it to improve this feature in pulps with low values of tear index (e.g., wastepaper pulps), i.e., as a “healing” pulp. However, it seems that this kind of work has not yet been conducted.

When pulp is obtained from IH stalks cut into 3–6 mm lengths the values of fibre length for such pulp do not indicate that it would cause application difficulties in the production of paper in industry and its further processing. However, such difficulties can be caused by the long IH bast fibres contained in such pulp. These difficulties may apply to the formation of paper. The observations made by the author of this publication suggest that the presence of such fibres in the pulp may impair this property of the paper. This effect of IH bast fibres could be limited by proper cutting of IH stalks. However, this can be difficult due to the tendency for the IH bast to separate from the woody core of IH stalks under mechanical forces, such as those applied to them when they are fed into the cutter. Other possibilities to reduce the adverse effect of IH bast fibres on paper formation are maintaining a small proportion of IH stalk pulp in the paper and an artificial reduction in the length of elementary fibres in IH bast, e.g., to the level of fibre length in kenaf bast, which involves the need to breed a new variety of hemp.

The yield of kraft pulps from the IH woody core can be described as having an intermediate value (i.e., approx. 47.5%) at kappa number 25, between that of birch pulp (53%) and pine pulp (44%) (Figure 4). Therefore, replacing a part of birch or pine wood with IH woody core should lead to a decrease in the kraft pulp yield from a mixture with the first of the listed wood species and an increase in the case of a mixture with the second one. Replacing part of the birch or pine wood with IH woody core is less favourable in terms of pulp yield from the FRM mixes than replacing part of the wood with IH stalks [101,128]. 

From the application point of view of the IH woody-core kraft pulp evaluation its ability to dewater on the paper machine screen and the influence of the short length and thin cell wall fibres on the properties of paper should be considered. The determination of the properties of IH woody-core pulp indicates that it should dewater on the paper machine wire worse than pulps made of birch and pine (Shopper-Riegler freeness in unbeaten state: 28 °SR vs. 13 and 14 °SR, respectively) and give the paper high breaking strength, light scattering ability (opacity), and density in handsheets but low specific volume (bulk) and tear resistance [125]. These results are due to the very high content of fibres in 1 g of pulp (32 mln), the short fibre length (0.5 mm), and the thin cell wall of the fibres (Table 4) [101]. This type of pulp is suitable for producing thin papers that require high opacity and tensile strength (e.g., bible paper or paper for cash registers). However, other types of paper, e.g., for printing and packaging, may require a larger bulk to obtain greater stiffness and fibre length and cell wall thickness to obtain greater tear resistance, which IH woody-core pulp cannot provide. Therefore, this type of pulp is less versatile than IH stalk pulp.

When evaluating the pulp yield from IH bast fibres, it can be stated that when the proper production method of kraft pulp from these fibres is used, it significantly exceeds the yield of such pulps from wood. With the processing of these fibres consisting only of oxygen delignification and ECF bleaching, a fully bleached pulp can be obtained from these fibres with a yield of as much as 85% [124]. 

As for the properties of IH bast fibre pulp, one can state that it is characterised by high tear resistance and high bulk but a low breaking length and low ability to scatter light, which results from the low number of fibres in 1 g of this pulp, the low content of hemicelluloses (dominance of rigid cellulose–cellulose connections in the structure of the paper), and the relatively thick cell walls of these fibres. As in the case of pulp from cut IH stalks, in the case of IH bast fibres pulp, there is also a problem with the uneven paper formation of paper produced from this pulp. The main reason for this is probably the large differences in the lengths of the fibres [101,125]. 

The high bulk and tear strength of bleached IH bast fibre pulp, as well as the high α-cellulose and low hemicellulose content of this pulp, make it a potential fibrous material for the production of high-bulk specialty papers (e.g., filter papers), improvements in the bulk and tear index of pulps characterised by low values of these properties (e.g., wastepaper pulps), and chemical processing (as dissolving pulp).

Considering the data mentioned above, it seems that the best way to introduce IH into EKPMs is to replace part of the wood with IH stalks, mainly because pulp from these stalks enables one to obtain the most balanced paper properties and properties similar to those of kraft pulps, especially pine. In addition, this allows one to increase the pulp yield from a mixture of wood and IH stalks by 2–3% at the 20% share of IH stalks in the mixture [128,129].

### 5.4. Competition from Other Nonwood FRMs

#### 5.4.1. Cereal Straw

One of the most obvious alternatives to wood for producing papermaking pulps in Europe is straw from cereals, mainly wheat, rye, and triticale. In terms of availability, it is much better than IH. For example, it has been reported that the cultivation of consumption cereals in Poland in the last 20 years enabled the country to obtain from 23 to 35 million t of straw from cereals (including corn and rape) per year, a significant part of which has been used in agriculture for bedding, fodder, and soil fertilisation, resulting in a surplus of 25–32% of this amount [89,135,136]. However, straw is also considered an alternative FRM for generating thermal energy in the form of bales, briquettes, and pellets. Nevertheless, the use of even several thousand tons of such straw for papermaking purposes could significantly reduce the interest in using industrial IH stalks for this purpose. Cereal straw can also be considered a more competitive FRM than IH in terms of pulp properties due to the lack of long IH fibres, which, as mentioned earlier, can cause spinning problems and deteriorate the paper formation of IH pulp. So far, however, the use of cereal straw for papermaking purposes in EKPMs has been very small. This is mainly due to the difficulties encountered in the joint processing of wood and cereal straw, such as faster pulping than wood, larger number of fine cells with large specific surfaces, and a much larger amount of mineral substances in it that pass into the post-pulping liquor and cause difficulties in the stage of its processing in the alkali recovery department of kraft pulp mills. In this regard, IH stalks fare better because they contain fewer high-surface-area cells and mineral substances than cereal straws.

In terms of minimising problems with the spinning of IH bast fibres at the stage of processing IH stalks into unbleached and bleached kraft pulps, the formation of paper obtained from IH stalk pulp and the too-low tear resistance of IH woody-core pulp, the competitiveness of these stalks, and the IH woody core in relation to cereal straw, these would certainly be improved by a significant reduction in the length of IH bast fibres in the IH stalks and an increase in this feature of fibre in the IH woody core. However, this requires breeding a new type of IH, which one might call a papermaking variety.

#### 5.4.2. Miscanthus × Giganteus

*Miscanthus* × *giganteus* can be considered one of the competitors to IH when used for papermaking purposes. Although it is a plant originating from Asia, it can be cultivated with good results in Europe. Its advantages over IH are as follows: it has approximately similar high stalk yield per hectare of cultivation as IH; there is no need for annual sowing (gaining biomass is possible for 15–20 years after establishing the plantation); and it lacks the long bast fibres and the related runnability processing problems in EKPMs and problems related to paper properties [137,138]. As far as Poland is concerned, the problem with *Miscanthus*, from a papermaking point of view, is the small area of cultivation of this plant (in 2011, about 4000 ha [139]). The usefulness of *Miscanthus* for papermaking is also reduced by its frequent use for obtaining thermal energy (e.g., pellet production) due to fewer problems with grinding of *Miscanthus* straw than IH stalks [140] and the high content of leaves (approx. 26–36%) less useful for producing paper pulp than stalks [141]. However, the plant appears more competitive than IH in terms of paper properties due to the lack of long bast fibres and higher fibre length in the woody part of the stem than in the case of IH.

#### 5.4.3. Virginia Mallow (Sida Hermaphrodita)

Virginia mallow, similar to *Miscanthus × gigantheus*, is a perennial plant (use period of 15–20 years). It is resistant to frost and drought, has low fertilisation requirements, and has a somewhat lower ash content than cereal straws (2.3–2.7%). From the point of view of papermaking, significant features of V. mallow are the height of its stems, sometimes even up to approximately 4 m (in Poland, 2.2–2.9 m), and its considerable width, from 5 to even 33 mm. In the climatic conditions of Poland, yields of V. mallow per hectare of cultivation have amounted to approximately 9–22 t (in Poland 7–15 t) of stalks and leaves at a humidity of 23–30% and an unknown content of leaves in biomass [142,143]. Preliminary studies in Poland on the length distribution of V. mellow fibres in kraft pulp showed that the average length of its wood pulp fibres is low, about the same as in IH woody-core pulp (i.e., approximately 0.5 mm). The average length of bast pulp fibres in this plant is about 2 mm, and the range of fibre lengths is from 0.5 to 6 mm. The share of phloem in the lower part of the stem is approximately 14%.

#### 5.4.4. Kenaf (Hibiscus Cannabinus)

Kenaf is a plant that grows in countries with a warm climate and high rainfall. During 6–8 months of growth, the kenaf plant reaches a height of 0.90–6.0 m and a diameter of 2.5–5.0 cm. With dense planting, the kenaf stems do not produce much branching. The annual harvest of kenaf straw is large, even up to 30 t/ha. In research conducted by Grabowska et al. [82], kenaf plantations established in Poland (and, therefore, in the northern temperate climate zone) gave a similar amount of biomass (stalks and leaves) per hectare of cultivation as IH of the Białobrzeskie variety, i.e., about 14 t. However, kenaf turned out to be much more susceptible to adverse weather conditions than IH. This was manifested by the inhibition of this plant’s growth during periods of low temperatures and lack of rainfall. However, research on the possibility of growing kenaf in the temperate climate zone is worth continuing because this plant has an advantage over IH regarding its bast fibre pulp down into single elementary fibres, which are characterised by a range of fibre lengths (0.2–6 mm), much closer to the range of hardwood and coniferous wood fibres [144,145] than IH fibres (2–55 mm).

As a result, kenaf is expected to have better processability and better paper formation than IH stalks in devices designed for wood processing in EKPMs and semi-chemical and mechanical pulp mills.

### 5.5. Economic Factors

#### 5.5.1. Price Competitiveness of IH FRMs

Table 4 contains the prices of IH FRMs, pulpwood, and other nonwood FRMs expressed in EUR. 

**Table 4:** Prices (in EUR) of IH FRM, pulpwood, and other nonwood FRMs (without transport costs).

FRM		Dry Hemp Stalks without Leaves	Dry Hemp Hurds (Woody-Core)	Dry Hemp Bast Fibres	Birch and Pine Pulpwood	Cereal Straw (Wheat)	*Misc. gigant*. Stalks	V. Mellow Stalks
Source	Year		1st, 2nd or 3rd Class of Purity	-	-	-	-	-
[146]	2011	122	275	782	-		-	-
Various references	2013–2023	90–135 ^(3)^	79, 213 and 382 ^(1)^; 463 ^(8)^	514–771^(8)^	160– 170 ^(2)^	41 ^(7)^–51 ^(4)^	100–120 ^(5)^	107 ^(6)^

^(1)^ [147] (In the case of hemp, the following was assumed: 8% VAT, dryness of raw materials 84%, leaf content in hemp straw 12.5%); ^(2)^ [148], ^(3)^ [149] ^(4)^ [150] ^(5)^ [151] (calculated assuming a price of EUR 78 Euro per ton of dry *Miscanthus* straw, VAT of 8%, and 30% share of leaves in this straw); ^(6)^ [152] (calculated assuming a price EUR 63 per ton of dry V. mellow straw, 30% share of leaves in this straw, and dryness of raw materials of 84%), ^(7)^ [153], ^(8)^ [154].

Table 4 shows a lower price for IH stalk straw than cleaned IH woody-core and bast fibres. The high costs of the latter two FRMs probably result from the increased demand for them in the production of hemp–lime–cement concrete and the amount of work needed to separate IH bast fibres from the stalks, with their low share in this stalk. So, regarding price, IH stalks were the best FRM for papermaking among the IH FRMs. As shown in Table 2, their price per ton is even lower than that of wood in Poland. Unfortunately, IH stalks are two-times more expensive than wheat straw, but their price can be comparable to the price of *Miscanthus* × *giganteus* stalks and Virginia mallow stalks.

#### 5.5.2. Other Costs Related to the Production of Papermaking Pulps from IH FRMs

In addition to the purchase price of hemp stalks, the costs of their transport to the pulp mill, storage, and processing into kraft pulp (chemicals, electricity, labour) should be taken into account in the analysis of the cost of producing one ton of pulp from this FRM.

Cost of transportation to the pulp mill. The cost of transporting any cargo is charged per kilometre of transportation. Therefore, it will depend on the distance between the hemp plantation and the pulp mill. At the same time, the higher the density of the transported material, the lower the cost. For example, it is known that the cost of transporting cereal straw to a pulp mill is higher than the cost of transporting wood, which is denser than straw. In the case of hemp straw, the way to increase the load density is to bale it densely. However, the cost of transporting it will be slightly higher than that of wood due to the lower density of baled hemp straw compared to wood. It seems, however, that the cost of transporting hemp straw should not be higher than the cost of transporting used corrugated cardboard to the pulp mill, which is much more voluminous than wood. After all, the use of used corrugated cardboard for paper purposes is not abandoned because of the reason of higher cost of transport to the pulp mill than wood.

The cost of storing the FRM. Hemp is an annual plant, so ensuring the supply of raw material needed for the pulp mill operation from the moment of harvest in a given year to the moment of harvest in the following year would require the collection of an appropriate amount. For a pulp mill processing around 2000 t of raw material per day, this stock would have to be 320 days × 2000 t = 640,000 t. Of course, this is a purely hypothetical case because, currently, there is no need to switch a pulp mill, whose production is based on wood, entirely to production based on industrial hemp. The considerations should, at most, consider the possibility of a partial replacement of wood with hemp raw material in the event of its shortage or the implementation of the idea of producing paper products with a reduced share of wood fibres. For example, the realistic amount of wood that could be replaced with hemp at the beginning seems to be 5%. In this case, an industrial-scale pulp mill would have to accumulate a stock of 32,000 t of hemp stalks and bear the storage costs of this raw material. In the United States, the cost of storing kenaf over a six-month period was estimated at USD 15 per ton. This amount includes the construction of a warehouse, its maintenance, the depreciation of FRM, and fibre losses [155]. To avoid the costs of storing large amounts of hemp stalks in a pulp mill, an alternative solution could be their successive delivery from nearby farms where they would be stored. The possibility of using such a solution was proven in Spain, specifically with the Saica plant, which processes cereal straw in this system without major problems [156].

Chemical, electricity, and labour costs. Table 5 presents a comparison of the types and amounts of individual technological operations that hemp stalks would have to undergo, from raw material to bleached kraft pulps, in comparison with wood. 

These data indicate that the number of operations to be performed in the case of processing hemp stalks into bleached kraft pulp is the same as in processing wood into this type of pulp. The number of these operations could be reduced if hemp straw devoid of these plant parts was delivered to the pulp mill. On this basis, it can be assumed with a high probability that the amount of chemicals required to produce bleached pulp from hemp stalks will not be greater than for processing wood into this type of pulp. For this reason, the costs of energy and labour will also not be higher. As already mentioned, the costs of replacing some of the wood with cut hemp stalks may increase the problems associated with the processability of long hemp fibres in devices such as screen sorters. However, this problem may be limited by the joint processing of hemp stalks with birch and pine wood with a low share of hemp stalks in the mix (reduced concentration of bast fibres in the pulp suspension) and the availability of hemp varieties with fibre lengths more similar to the length of wood fibres in the future.

## 6. Conclusions

1. Currently, there are reasons to increase the scope of using IH pulps in European large-scale papermaking within the initiative of expanding the raw material base of this industry. This may contribute to limiting the felling of trees from natural forests for papermaking purposes, increase the use of wood for other non-papermaking applications, allowing for the retention of CO_2_ stored in wood for a longer time than in the case of paper and cardboard (e.g., furniture, roof trusses), and the introduction of papermaking products with a reduced content of fibres from natural forests on the market.

2. The use of IH for papermaking is favoured by the fact that IH is a well-known plant in Europe. It is well adapted to the conditions of the northern temperate climate zone and contains fewer mineral substances than cereal straw. Unfortunately, the cultivation area of this plant in Europe is much smaller than that of cereals, and its anatomical parts (bast fibres, woody-core) can be used for purposes other than papermaking, which may create additional competition for such applications of IH stalks compared to cereal straw and stalks of *Miskanthus* × *giganteus* and Virginia mallow.

3. The analysis of cultivation factors indicates that the disadvantages of IH as a potential FRM for papermaking are as follows: the need to register its cultivation, its small area, the need for annual sowing, and the lack of varieties with bast and woody-core fibre lengths more similar to wood fibre lengths. On the other hand, the advantages are as follows: no need to use plant protection products and herbicides, good plant resistance to low rainfall (deep root system), and a relatively high annual yield of stalks per hectare of cultivation.

4. From the point of view of papermaking, the following technological factors in applying IH in papermaking are noteworthy. The material and bulk density of IH stalks are more similar to wood than cereal straw. Obtaining kraft pulps from IH is easier than in the case of wood. There is the possibility of obtaining unbleached and bleached pulps from IH FRM using the method used in kraft pulping mills (favourable factors). Two types of fibres are present in IH stalks, and they are diametrically different in their dimensional properties and ability to bond with each other (unfavourable factor).

5. IH stalks are the best FRM for European large-scale papermaking as they are the cheapest among IH raw materials. Also, they can be processed more easily into bleached kraft pulp with a higher pulp yield than most hardwoods and softwoods, and they yield papers with properties more similar to those of wood pulps than pulps from hemp woody-core or hemp bast fibres.

6. In relation to IH, the analysis of the competitiveness of other NFRMs that grow well in Europe showed a higher competitiveness of wheat straw in terms of availability (hundreds of times higher) and price (2–3-times higher) compared to hemp stalks and a higher competitiveness of *Miscanthus* × *giganteus* stalks and wheat straw in terms of fractional composition of pulps from uncut material (fibre distribution). Unlike IH, these FRMs do not contain long bast fibres and far fewer short fibres (this is particularly evident in the case of *Miscanthus* × *giganteus*). In this type of comparison, the fibrous composition of the Virginia mallow can be described as intermediate because the pulp from its stems does not contain long bast fibres but contains a similar amount of short fibres as the pulp obtained from IH stalks. For this reason, *Miscanthus* × *giganteus* stalks, wheat straw, and Virginia mallow stalks can be considered more competitive than IH stalks in terms of compatibility with EKPM equipment and paper formation. In this aspect, the situation could be changed by having an industrial IH variety with a much shorter length of fibres in the IH bast and a slightly longer (e.g., by 0.2–0.3 mm) fibre length in the hemp woody core.

7. Easier processing of hemp stalks into unbleached kraft pulp (lower lignin content); a similar number of stages of processing this raw material into bleached kraft pulp; the possibility of reducing the costs of storing these stalks in the pulp mill through their successive transport from the plantation; and the possibility of reducing the share of hemp stalk fibres to a few per cent in suspensions of pulps flowing through the pulp mill installations make it possible to determine the possibility of keeping the production costs of pulps from wood/hemp stalks mixes at a level comparable to the production of such pulps from wood.

## Figures and Tables

**Table 1:** The content of chemical components in anatomical parts of IH stalks.

Component	IH Bast, ^a,b,c,j^	IH Bast Fibres ^a,d,e,f^	IH Woody-Core ^a,b,c,d,e,f^	IH Stalks ^a,g,h,i,k^
	% oven-dried FRM			
Cellulose	53.2–69.7	60–74	31.5–47.3	53.3–55.5
α-Cellulose	60.6–66.6	-	33.4–37.4	43.0–60.1
Holocellulose	81.2–87.4	90.0	72.0–72.3	72.8–83.5
Hemicelluloses	6.7–9.3	10–15	15.3–25.0	24.4
Lignin	3.3–10.0	2.9–3.8	18.8–25.3	12.4–21,8
Pectins	-	2.5–6.4	2–4	0.5–3.3
Extractives	1.2–13.4	1.4–12	1.2–15.9	0.6–3.8
Hot water extract	9.5–10.9	2.3	6.8	5.4–10.0
Ash	2.1–4.7	1.5–5.9	0.9–6.0	2.2–4.2

(a) [91]; (b) [92]; (c) [78]; (d) [93], (e) [94], (f) [95], (g) [61], (h) [96], (i) [97], (j) [98], (k) [99,100].

**Table 2:** Chemical characteristics of IH FRMs of the Białobrzeskie variety, as well as birch and pine wood [101].

	Component	Cellulose by Seifert	Lignin	Extractives	Ash	Hemicelluloses and Pectin ^(1)^
Kind of IH FRM						
		% oven-dried FRM				
Stalks		46.9	18.2	1.1	1.3	32.5
Woody-core		36.3	27.1	1.2	1.2	34.3
Bast fibres		71.7	4.0	0.9	1.5	21.9
Birch wood		42.6	20.4	2.8	0.3	33.9
Pine wood		45.5	28.5	1.6	0.4	24.0

^(1)^ Values calculated by subtracting the sum of cellulose, lignin, extractives, and ash contents from 100%.

**Table 3:** Properties of hemp stalks kraft pulp (Hs), hemp woody-core kraft pulp (Hw), and hemp bast fibre (Hbf) soda-oxygen-pulp [101] (all pulps bleached) [101].

Index (Unit)	Hs	Hw	Hbf
Number of fibres in 1 g of pulp, M/g	16.98	32.25	2.64
Length of fibre, mm	0.90	0.51	2.24
Fibre width (D), μm	22.8	20.5	23.7
Cell wall thickness (t), μm	1.67	0.95	5.46
F/C I (D/t)	13.6	21.6	4.3

**Table 5:** Comparison of the type and number of technological operations with separate processing of hemp stalks and wood into bleached kraft pulps.

O.n.	Hemp Stalks	Pine and Birch Wood
1	Separation of hemp leaves and inflorescences ^(a)^	Debarking
2	Cutting into sections, e.g., 0.3–0.6 cm	Cutting into chips
3	Kraft pulping, 15% o.d., FRM (other conditions are the same as in the case of wood processing	Kraft pulping using 22% and 17% o.d. FRM, respectively.
4	Oxygen delignification (the same conditions as for wood processing)	Oxygen delignification
5	Bleaching (the same conditions as for wood processing)	Bleaching

^(a)^ Only when hemp straw containing leaves and inflorescences is delivered to the pulp mill.

## Data Availability

The author of this work used collections of magazines (paper form) and legal electronic databases available at the Library of Lodz University of Technology.

## Abbreviations

BCE	before current era
ECF	Elemental Chlorine-Free
EKPM	European kraft pulp mills
EU	European Union
FRM	fibrous raw material
Hbf	hemp bast fibres
Hs	hemp stalks
Hw	hemp wood
IH	industrial hemp
NFRM	nonwood fibrous raw material
O.d.	ordinal number
THC	tetrahydrocannabinols
